# Risk Factors and Prevention Strategies for Chronic Postoperative Inguinal Pain After Laparoscopic Inguinal Hernia Repair: A Narrative Review

**DOI:** 10.7759/cureus.111537

**Published:** 2026-06-26

**Authors:** Dimitrios Bakaoukas, Dimitrios Filippou

**Affiliations:** 1 Surgery, Tzaneio General Hospital, Piraeus, GRC; 2 Anatomy, National and Kapodistrian University of Athens School of Medicine, Athens, GRC

**Keywords:** chronic postsurgical pain, inguinal hernia repair, laparoscopic, minimally invasive, prevention, risk factors

## Abstract

Inguinal hernias impact millions of people each year. One of the primary complications following inguinal hernia repair is chronic postoperative inguinal pain, which significantly affects patients' quality of life. While laparoscopic surgery has been shown to reduce the incidence of this complication, a considerable number of patients are still affected. Various risk factors have been identified, encompassing both patient-related and surgical factors. Investigating these risk factors is essential for enhancing our understanding of chronic postoperative inguinal pain and finding strategies for its prevention. A narrative review utilizing the PubMed and Scopus databases was conducted, employing the following search terms: chronic postsurgical pain AND inguinal hernia repair AND (risk factors OR prevention) AND (laparoscopic OR minimally invasive), which produced 50 articles for analysis. This article explores the risk factors associated with chronic postoperative inguinal pain following laparoscopic inguinal hernia repair, focusing on both patient-related and surgery-related aspects, as well as potential preventive measures to mitigate this issue. It ultimately highlights that, despite the identification of numerous risk factors and prevention strategies in existing literature, there remains a pressing need for more high-quality research, and challenges must be addressed to significantly lower the risk of this complication.

## Introduction and background

Inguinal hernia repair is one of the most prevalent surgical procedures, with estimates suggesting that over 20 million are performed each year [1]. While the conventional open Lichtenstein method is effective, laparoscopic techniques - especially the transabdominal preperitoneal (TAPP) and totally extraperitoneal (TEP) methods - are becoming increasingly popular due to their association with fewer complications, quicker recovery times, and lower incidence of chronic groin pain [2]. In the TAPP technique, the peritoneal cavity is entered laparoscopically, the peritoneum is incised, and the mesh is placed in the preperitoneal space, while in the TEP technique, the peritoneal cavity is avoided by creating a working space in the extraperitoneal plane [2].

Nevertheless, chronic postoperative inguinal pain (CPIP) remains a common and debilitating complication, affecting a substantial number of patients even after laparoscopic interventions [2]. Though definitions may vary, CPIP is generally described as moderate groin pain that interferes with daily activities and persists for at least three months following surgery [1]. This pain is believed to primarily arise from nerve injuries, such as those to the lateral femoral cutaneous nerve and the femoral and genital branches of the genitofemoral nerve, often due to improper tissue dissection and mesh placement [3]. Its occurrence can range dramatically from 0.7% to over 75%, influenced by definitions, follow-up durations, and measurement techniques [4]. For laparoscopic procedures, CPIP is estimated to affect 2.6%-13.8% of patients, with 9.5% experiencing pain one year after surgery and 2.5% needing treatment [3]. These figures underscore the severity of this complication, which can persist for years, even with minimally invasive surgical approaches.

Various factors, including younger age, female gender, and elevated body mass index (BMI), have been linked to a heightened risk of CPIP following laparoscopic repair [3]. Investigating these risk factors could enhance our understanding of CPIP and identify strategies to prevent it, ultimately improving the long-term quality of life for patients with inguinal hernias.

Several systematic reviews and meta-analyses have evaluated CPIP following laparoscopic inguinal hernia repair. However, many focused primarily on incidence rates, operative techniques, or mesh fixation methods. A comparative synthesis specifically examining both patient-related and surgery-related risk factors, together with preventive strategies, remains limited. Given the growing body of literature published in recent years, an updated review is warranted to provide a comprehensive overview of current evidence. 

The primary objective of this narrative review is to identify patient-related and surgery-related risk factors associated with CPIP following laparoscopic inguinal hernia repair. The secondary goal is to evaluate preventive strategies that may reduce CPIP rates and improve postoperative outcomes.

## Review

Methods and materials

This narrative review was conducted to identify patient-related and surgery-related risk factors associated with CPIP following laparoscopic inguinal hernia repair and to evaluate preventive strategies reported in the literature. The review was conducted according to the population, intervention, comparison, outcomes, and study (PICOS) framework. The population included adult patients undergoing laparoscopic inguinal hernia repair (TAPP or TEP). Interventions and exposures consisted of patient-related and surgery-related risk factors, as well as preventive strategies for CPIP. Comparators varied according to study design and included different patient characteristics, surgical techniques, mesh types, and fixation methods. Outcomes included incidence and severity of CPIP ≥ 3 months postoperatively, quality of life, treatment requirement, recurrence, and postoperative complications. Evidence was derived from randomized controlled trials (RCTs), cohort studies, observational studies, guidelines, case controls, narrative reviews, systematic reviews, and meta-analyses in order to provide a broad overview of the available literature. A thorough investigation was conducted using literature obtained from the PubMed and Scopus databases. The search strategy, including databases, keywords, publication period, and search outcomes, is summarized in Table 1. The keywords utilized were as follows: chronic postsurgical pain AND inguinal hernia repair AND (risk factors OR prevention) AND (laparoscopic OR minimally invasive). A filter was applied to include studies published between 2010 and 2026. The inclusion criteria applied were studies evaluating chronic postsurgical pain after laparoscopic inguinal hernia repair and studies with appropriate and not immediate postoperative pain follow-up, while the exclusion criteria included studies focused solely on open inguinal hernia repair, studies evaluating only immediate postoperative pain, articles without full text access, studies with insufficient patient sample size, articles containing irrelevant material, and studies with inconclusive findings.

**Table 1:** Search strategy

Database	Keywords/Search strategy	Publication filter	Articles identified
PubMed	chronic postsurgical pain AND inguinal hernia repair AND (risk factors OR prevention) AND (laparoscopic OR minimally invasive)	2010-2026	271
Scopus	chronic postsurgical pain AND inguinal hernia repair AND (risk factors OR prevention) AND (laparoscopic OR minimally invasive)	2010-2026	241
Total	-	-	512

Two reviewers (DB and DF) independently screened the titles and abstracts of all records identified through the database search. Full-text articles of potentially eligible studies were subsequently assessed independently according to the predefined inclusion and exclusion criteria. Any disagreements regarding study eligibility or data extraction were resolved through discussion and consensus. Data extracted from the included studies included study design, sample size, follow-up duration, CPIP definition, pain assessment methods, identified risk factors, and reported prevention strategies.

Initially, 512 articles were found, with 271 sourced from PubMed and 241 from Scopus. Prior to screening, one duplicate was eliminated from the Scopus database, and 405 were discarded for having irrelevant titles. A total of 106 articles were reviewed, 96 coming from PubMed, and 17 of these were similar articles within the same database. Out of these 106 articles, 1 was excluded due to lack of full access; 12 were not relevant as they focused solely on open procedures rather than laparoscopic ones; 20 were dismissed because their postoperative pain follow-up period was immediate rather than three months, which is critical for CPIP definition; three had insufficient patient samples and were not considered; 16 contained irrelevant material; and four yielded inconclusive findings and were therefore not usable for this article. Ultimately, 50 studies were included in the review and fulfilled the inclusion criteria, which were studies evaluating CPIP after laparoscopic inguinal hernia repair and studies with the appropriate follow-up time. Due to heterogeneity in study design, CPIP definitions, follow-up duration, and outcome assessment tools, quantitative meta-analysis was considered inappropriate, and a narrative synthesis was performed.

Results

Characteristics of the Included Studies

The characteristics and primary focus of the included 50 studies are summarized in Table 2. Of the 50 articles reviewed, 21 studies focused on patient-related risk factors, 22 on surgery-related aspects, 23 on preventing CPIP, and 2 were dedicated to discussing limitations. This compilation included studies, 27 (54%) cohort studies (7 prospective and 20 retrospective), 7 (14%) systematic reviews/meta-analyses, 8 (16.0 %) RCTs, 3 (6%) narrative reviews, 2 (4.0%) were pilot observational studies, 1 (2.0%) was an observational study, 1 (2.0%) was a case-control study, and 1 (2.0%) comprised the international groin hernia management guidelines. Additionally, 46 (92.0%) of these studies came from PubMed, and four (8.0%) from Scopus, corresponding to a PubMed-to-Scopus ratio of 11.5:1. The included studies primarily investigated risk factors and preventive strategies associated with CPIP following laparoscopic inguinal hernia repair. Reported CPIP incidence varied considerably across studies due to differences in definitions, follow-up duration, and assessment methods. Several studies identified both patient-related and surgery-related factors associated with CPIP, while others evaluated interventions aimed at reducing its occurrence.

**Table 2:** Characteristics and primary focus of the included studies NRS: Numerical Rating Scale; sf-IPQ: Short-form Inguinal Pain Questionnaire; IPQ: Inguinal Pain Questionnaire; CCS: Carolina Comfort Scale; EuraHS: European Registry for Abdominal Wall Hernias Survey; VAS: Visual Analog Scale (VAS); SOMS: Surgical Outcomes Measurement System (SOMS); VRS: Verbal Rating Scale

No.	Authors	Year	Study design	Database	Main focus	Follow-up duration	Pain assessment tool	CPIP definition	Sample size
1	HerniaSurge Group	2018	Guideline	PubMed	Patient risk-related factors, surgery-related risk factors, prevention	Not applicable	No single assessment tool specified	≥3 months	N/A
2	Naim et al.	2026	Systematic review and meta-analysis	PubMed	Surgery-related risk factors, prevention	Variable	Various	Variable	7 studies
3	Sato et al.	2025	Pilot observational study	PubMed	Prevention	3 months	NRS	NRS ≥ 3 at 3 months	188 TAPP procedures (222 hernias); AI trained on 62 cases (73 hernias)
4	Novik et al.	2025	Retrospective cohort study	PubMed	Patient risk-related factors, prevention	12-15 months	sf-IPQ	sf-IPQ-based chronic pain at 1 year	15,360 eligible patients (10,525 respondents analyzed)
5	Zhou et al.	2025	Retrospective cohort study	PubMed	Patient risk-related factors, prevention	>1 year	Numeric Rating Scale (0-10)	>1 year, no formal definition reported	380 patients
6	Stolt et al.	2025	Prospective cohort study	PubMed	Patient risk-related factors	1 year for CPIP, 4.9 years for recurrence	IPQ	IPQ score 4-7 at 1 year	65,749 patients
7	Alaverdyan et al.	2024	Systematic review and meta-analysis	PubMed	Patient risk-related factors, surgery-related risk factors, prevention, limitations	Variable	Various	Variable	303 studies included
8	Bakker et al.	2019	Prospective cohort study	PubMed	Patient risk-related factors	3 months, 1 year, 2 years	NRS, IPQ, CCS	Pain >3 months after TEP repair	867 completed follow-up
9	Iyer et al.	2025	Retrospective cohort study	PubMed	Patient risk-related factors	1 year	EuraHS	1 year, no formal definition reported	1,582 patients
10	Luk et al.	2020	Retrospective cohort study	PubMed	Patient risk-related factors	Median 42 months	No validated pain scale specified	No formal CPIP definition reported	84 female patients
11	Zhong et al.	2024	Meta-analysis and trial sequential analysis	PubMed	Patient risk-related factors	Variable	Various	Variable	6 studies, 1,119 female patients
12	Köckerling et al.	2021	Retrospective cohort study	PubMed	Patient risk-related factors	1 year	Herniamed Registry questionnaire	Pain at rest, pain on exertion, or pain requiring treatment at 1 year	98,321 patients
13	Kim et al.	2021	Retrospective cohort study	PubMed	Patient risk-related factors, prevention	6 months	VAS	Pain persisting for more than 3 months after surgery	960 patients
14	van Hout et al.	2018	Retrospective cohort study	PubMed	Patient risk-related factors	12 months	No standardized tool reported	Pain >3 months after surgery	152 patients
15	Aasvang et al.	2010	Prospective cohort study	PubMed	Patient risk-related factors	6 months	NRS	No formal CPIP definition reported	464 patients
16	Widder et al.	2023	Retrospective cohort study	PubMed	Patient risk-related factors	Not specified	EuraHS QoL questionnaire	Pain ≥3/10 (VAS/NRS) lasting >3 months postoperatively	344 patients
17	van den Dop et al.	2022	Prospective cohort study	PubMed	Patient risk-related factors	1 month, 1 year, 2 years	VRS	≥3 months	4.016 patients
18	Vad et al.	2019	Prospective cohort study	PubMed	Patient risk-related factors, prevention	6 months	NRS	Not reported	2508 patients
19	Patel et al.	2017	Prospective cohort study	PubMed	Patient risk-related factors, prevention	3 weeks, 6 months, 1 and 2 years	Short Form-36, SOMS and CCS	Not reported	482 patients
20	Rosenberger et al.	2022	Review	Scopus	Patient risk-related factors, prevention	Variable	Various	≥3 months	N/A
21	Martin et al.	2021	Retrospective Cohort Study (Registry-based)	Scopus	Patient risk-related factors, prevention	12 months and 5 years	sf-IPQ	No formal CPIP definition reported	4308 patients
22	Brenner et al.	2021	Review	Scopus	Patient risk-related factors	N/A	N/A	No formal CPIP definition reported	N/A
23	Kalliomäki et al.	2016	Case-control study	Scopus	Patient risk-related factors	N/A	IPQ	No formal CPIP definition reported	100 patients
24	Bittner et al.	2025	Retrospective cohort study	PubMed	Surgery-related risk factors	Not reported	Not reported	Not reported	222,487 patients
25	Ma et al.	2025	Retrospective cohort study	PubMed	Surgery-related risk factors	Not reported	VAS	Not reported	163 male patients
26	Ruze et al.	2019	Randomized controlled trial	PubMed	Surgery-related risk factors	7 days, 1 and 3 months	VAS	Not reported	159 patients
27	Li et al.	2020	Randomized controlled trial	PubMed	Surgery-related risk factors	12 months	VAS	≥3 months	70 patients
28	Mehrotra et al.	2025	Retrospective cohort study	PubMed	Surgery-related risk factors	6 months	Not reported	Not reported	136 patients
29	Zhu et al.	2019	Randomized controlled trial	PubMed	Surgery-related risk factors	7 days, 1, and 3 months	VAS	≥3 months	60 patients
30	Kane et al.	2018	Retrospective cohort study	PubMed	Surgery-related risk factors	21.6 ± 23.8 months	Not reported	Not reported	231 patients
31	Seefeldt et al.	2025	Randomized controlled trial	PubMed	Surgery-related risk factors	6 months and 2 years	VAS	No formal CPIP definition reported	491 patients
32	Bakker et al.	2021	Meta-analysis	PubMed	Surgery-related risk factors	3 to 60 months	Various	Variable	2909 patients
33	Burgmans et al.	2016	Randomized controlled trial	PubMed	Surgery-related risk factors	2 year	NRS	Not reported	950 patients
34	Aydin et al.	2025	Retrospective cohort study	PubMed	Surgery-related risk factors	1 day, 1 month, 3 months	VAS	≥3 months	155 patients
35	Zarras et al.	2025	Retrospective cohort study	PubMed	Surgery-related risk factors	1 year	Not reported	Not reported	3,098 patients
36	Yildirim et al.	2018	Retrospective cohort study	PubMed	Surgery-related risk factors	6 months	VAS	Not reported	303 patients
37	Meshkati Yazd et al.	2023	Randomized controlled trial	PubMed	Surgery-related risk factors, prevention	1, 3, and 6 months	VAS	Not reported	100 patients
38	Jiang et al.	2024	Meta-analysis	PubMed	Surgery-related risk factors, prevention	Variable	Various	Variable	9 RCTs involving 1,879 patients
39	Etele et al.	2020	Retrospective cohort study	PubMed	Surgery-related risk factors, prevention	1 and 3 months	Pain Detect Questionnaire	≥3 months	101 patients
40	Kitching et al.	2025	Meta-analysis	PubMed	Surgery-related risk factors, prevention	Variable	VAS	Variable	18 RCTs involving 2319 patients
41	Habib Bedwani et al.	2025	Meta-analysis	PubMed	Surgery-related risk factors, prevention	Variable	Various	Variable	15 RCTs involving 2109 patients
42	Köckerling et al.	2024	Retrospective cohort study	PubMed	Surgery-related risk factors	1 year	Not reported	Not reported	83768 patients
43	Vad et al.	2022	Prospective cohort study	PubMed	Prevention	6 months	NRS	NRS ≥2 at 6 months postoperatively	2,508 patients
44	Rey Chaves et al.	2024	Retrospective cohort study	PubMed	Prevention	1 year	VAS	≥3 months	296 patients
45	Nageeb et al.	2026	Randomized controlled trial	PubMed	Prevention	1 month	VAS	Not reported	146 patients
46	Xu et al.	2024	Retrospective cohort study	PubMed	Prevention	3 months	VAS	≥3 months	134 patients
47	Liu et al.	2020	Randomized controlled trial	PubMed	Prevention	3 months	VAS	Not reported	103 patients
48	Zygomalas et al.	2024	Pilot observational study	PubMed	Prevention	N/A	N/A	N/A	N/A
49	Mita et al.	2024	Observational study	PubMed	Prevention	N/A	N/A	N/A	N/A
50	Molegraaf et al.	2017	Review	PubMed	Limitations	N/A	Various	Variable	48 studies

Outcomes

The principal outcomes reported in the included literature are summarized in Table 3. Considerable variability was observed in CPIP incidence and severity due to differences in study design, follow-up duration, and assessment methods. Nevertheless, CPIP consistently demonstrated a negative impact on patient quality of life and, in a subset of patients, required further treatment.

**Table 3:** Summary of key outcomes reported in the included literature CPIP: Chronic Postoperative Inguinal Pain; NRS: Numerical Rating Scale; VAS: Visual Analog Scale

Outcomes	Summary of findings
CPIP incidence	2.6%-13.8%
CPIP severity	Variable according to NRS/VAS
Quality of life	Reduced in CPIP patients
Treatment requirement	Approximately 2.5% required treatment
Recurrence	Conficting evidence
Complications	Seroma, urinary retention, hematoma, etc.

Patient-Related Risk Factors Associated With CPIP

Patient-related risk factors associated with CPIP are summarized in Table 4. Patient-related factors associated with CPIP identified in the included studies included younger age, elevated BMI, female sex, femoral hernias, excision of the round ligament, high American Society of Anesthesiologists (ASA) grade, elevated preoperative European Registry for Abdominal Wall Hernias Survey (EuraHS) quality-of-life scores, smaller hernia size, bilateral hernia repair, recurrent hernias, medial hernias compared with scrotal hernias, smoking, benign prostatic hyperplasia (BPH), atypical symptoms and elective surgery for clinically undetectable hernias, preoperative pain, postoperative pain, postoperative analgesic use, sensory abnormalities in the groin at six months postoperatively, heavy lifting (> 1,000 kg/day), low income, poor general health and diminished quality of life, diabetes, hypertension and other comorbidities, lack of preoperative optimism, anxiety and depression, sleep deprivation, alterations in the gut microbiome, and genetic predisposition.

**Table 4:** Patient-related risk factors associated with CPIP BMI: Body Mass Index; ASA: American Society of Anesthesiologists; BPH: Benign Prostate Hyperplasia; CPIP: Chronic Post-inguinal Pain

Risk factor	Supporting studies	Contradictory/negative studies
Younger age	4, 5, 6, 7	8
High BMI	4, 5, 7, 9	0
Female sex	7, 9	0
Femoral hernias	4, 9	0
Excision of round ligament	9	10, 11
High ASA grade	4, 6, 7, 9	0
Elevated preoperative quality of life scores (EuraHS)	4, 6, 7, 9	0
Smaller hernia sizes	4, 6, 7, 9	0
Bilateral hernia repair	4, 6, 7, 9	0
Recurrent hernias	4, 6, 7, 9	0
Medial hernias compared to scrotal hernias	12	0
Smoking	5	0
BPH	13	0
Atypical symptoms and elective surgery for clinically undetectable hernias	14	0
Preoperative pain	1, 7, 15, 17	0
Postoperative pain	1, 7, 15, 17	0
Postoperative analgesics use	17	0
Sensory abnormalities in the groin by six months post-surgery	1, 15	0
Lifting heavy weights > 1000 kg/ day	18	0
Low income	5	0
Poor general health and diminished quality of life	19	0
Diabetes, hypertension, and comorbidities	7	0
Lack of preoperative optimism, anxiety and depression	1, 20	0
Sleep deprivation	21	0
Gut microbiome	22	0
Genetic component	1, 23	0

Surgery-Related Risk Factors Associated With CPIP 

Surgery-related factors associated with CPIP are summarized in Table 5. The factors identified in the included studies are composed pf open procedures, lack of surgeon experience, absence of a dedicated hernia center, postoperative complications, hospital volume, hernia sac management techniques, use of sutures to obliterate dead space, direct closure of hernia defects using barbed sutures, peritoneal flap closure, mesh type (biological versus synthetic and lightweight versus heavyweight meshes), slit versus non-slit mesh configuration, mesh fixation versus non-fixation, mechanical versus glue fixation, acrylate versus fibrin glue fixation, and mesh pore size.

**Table 5:** Surgery-related risk factors associated with CPIP CPIP: Chronic Post-inguinal Pain

Surgery related risk factors	Supporting studies	Contradictory/negative studies
Open procedures	2	0
Absence of surgeon experience	1, 7	0
Absence dedicated hernia center	1, 7	0
Postoperative complications	1, 7	0
Low-volume hospitals (associated with decreased CPIP risk)	24	0
Hernia sac management	0	25, 26, 27
Use of sutures to obliterate dead space	0	28
Direct closure of hernia defects using barbed suture	0	29
Peritoneal flap closure	0	30
Biological meshes compared to synthetic meshes	0	31
Lightweight meshes compared to heavyweight meshes	0	7, 32, 33
Slit versus non-slit meshes	36	34, 35
Mesh fixation compared to no fixation	38, 39	37
Mechanical fixation compared to glue fixation	40, 41	0
Acrylate glue compared to fibrin glue	7	0
Size of mesh pores	0	42

Prevention Strategies for CPIP Risk Reduction

Preventive strategies for reducing CPIP risk are summarized in Table 6. The preventive methods identified in the included studies included risk stratification and preoperative prediction models, smoking cessation, weight loss, management of comorbid conditions, improvement of overall health status, treatment of BPH, avoidance of heavy lifting (> 1,000 kg/day), management of sleep deprivation and psychological factors, laparoscopic repair rather than open repair, avoidance of mesh fixation, use of fibrin glue fixation, use of self-fixating meshes instead of staple fixation, performance of surgery by experienced surgeons, fascia transversalis plication in direct inguinal hernias, preperitoneal space dissection without electrocoagulation, use of monopolar energy versus blunt dissection during TEP repair, and artificial intelligence-assisted anatomical landmark recognition.

**Table 6:** Prevention strategies for CPIP following laparoscopic inguinal hernia repair AI: Artificial intelligence; CPIP: Chronic Post-inguinal Pain

Prevention strategies	Supporting studies	Reported effect
Risk stratification and preoperative prediction models	45	Potential benefit
Smoking cessation	5	Potential benefit
Weight loss	4, 5, 7, 9	Potential benefit
Management of comorbid conditions	7	Potential benefit
Improvement of overall health	19	Potential benefit
Treatment of Benign Prostatic Hyperplasia	13	Potential benefit
Avoiding heavy lifting (over 1000 kg daily)	18	Potential benefit
Managing sleep deprivation	21	Potential benefit
Managing psychological issues	1, 20	Potential benefit
Laparoscopic repair compared to open repair	2	Associated with lower risk of CPIP
Avoiding mesh fixation	7, 38, 39	Associated with lower risk of CPIP
Fibrin glue fixation	7, 40, 41	Associated with lower risk of CPIP
Self-fixating meshes compared to stable fixation	44	Associated with lower risk of CPIP
Experienced surgeon	1, 7	Associated with lower risk of CPIP
Fascia transversalis plication in direct inguinal hernias	45	Potential benefit
Preperitoneal space dissection without electrocoagulation	46	Associated with lower risk of CPIP
Monopolar energy compared to blunt dissection in TEP repair	47	No reported benefit
Use of AI for anatomical landmark recognition	3, 48, 49	Potential benefit

Discussion

Numerous risk factors have been identified in relation to chronic post-inguinal pain (CPIP) following laparoscopic inguinal hernia repair, categorized into patient-specific and surgery-related elements.

Patient-Related Factors

Research indicates that younger age tends to elevate the risk of CPIP, potentially linked to psychological factors and a heightened immune and neural response to surgical procedures [4-7]. A notable exception is a prospective study involving 867 patients undergoing TEP repair, which found no relationship between age and CPIP [8]. Body mass index (BMI) is another contributor to CPIP, likely through inflammation caused by excess fat tissue, mechanical strain from added weight, and surgical complexities [4,5,7,9]. Female gender is also a significant risk factor, with observations suggesting laparoscopic methods do not effectively mitigate CPIP in women [7]. This may be attributed to increased postoperative anxiety and depression, inadequate pain management, diminished pain inhibition, hormonal influences, and conditions such as pelvic floor dysfunction [7,9]. Additionally, the occurrence of femoral hernias - which can lead to CPIP - is more common in females [4,9]. Some researchers have suggested that excising the round ligament during surgery could contribute to CPIP in females [9]; however, two studies indicate that such excision does not affect CPIP incidence [10,11].

A higher grade on the American Society of Anesthesiologists (ASA) physical status scale, elevated preoperative quality of life scores (EuraHS), smaller hernia sizes, bilateral repairs, and recurrent surgeries also increase CPIP risk [4,6,7,9]. The anatomical changes and tension on tissues caused by initial surgeries may predispose recurrent hernias to CPIP [7]. Köckerling et al. noted that medial hernias have an elevated risk of chronic discomfort compared to scrotal types [12]. Smoking, likely due to compromised tissue healing, along with benign prostatic hyperplasia (BPH) from elevated intra-abdominal pressure during urination, also heightens CPIP risk [5,13]. van Hout et al. emphasized that patients exhibiting atypical symptoms before surgery and those undergoing elective procedures for clinically undetectable hernias diagnosed via ultrasound are at increased risk for CPIP [14]. Further, preoperative pain, substantial postoperative pain, use of analgesics after surgery, and sensory abnormalities in the groin by six months post-surgery have been linked to higher CPIP rates [1,7,15,16]. Research by Alaverdyan et al. has shown that preoperative pain can involve compression neuropathy, possibly resulting in irreversible nerve damage [7]. Notably, van den Lop et al. discovered that postoperative pain at one month acts as a stronger predictor for CPIP than pre-surgery pain [17]. Vad et al. highlighted that lifting heavy weights (over 1,000 kg/day) correlates with CPIP incidence [18], while Zhou et al. found a relationship between low income and higher CPIP risk [5]. Patel et al. pointed out that poor general health and diminished quality of life significantly predict CPIP at six months [19]. Alaverdyan et al. also found links between chronic pain, diabetes, hypertension, and comorbidities, although not specifically in the context of inguinal hernia repairs [7].

Psychological variables appear influential in developing CPIP. A lack of preoperative optimism, along with anxiety and depression, increases the likelihood of CPIP [1,20]. Sleep loss contributes to both the incidence and severity of CPIP [21]. Brenner et al. [22] suggested that the gut microbiome might affect CPIP rates by modulating inflammation and pain responses, with alterations caused by stress linked to chronic postoperative discomfort. Genetic predisposition has also been noted, with several genes and polymorphisms, such as human leukocyte antigen, DQ Beta 1 (DQB1 HLA) and tumor necrosis factor alpha (TNF-α), associated with CPIP [1,23].

Surgical Factors

As previously mentioned, open surgical procedures elevate the risk of CPIP [2]. In laparoscopic inguinal hernia repairs, both TAPP and TEP approaches yield comparable outcomes regarding chronic pain [2]. Factors such as surgeon experience, the presence of a dedicated hernia center, and postoperative complications significantly increase the risk of CPIP [1,7]. Bittner et al. noted that CPIP rates were lower in laparoscopic repairs performed at low-volume hospitals, potentially due to stronger patient-surgeon connections [24].

Concerning surgical techniques, multiple studies indicate no significant impact on CPIP incidence based on hernia sac management [25-27]. Nonetheless, Ma et al. found that a specific method - lateral stump fixation following midline transection - resulted in lower seroma rates [25]. Ruze et al. reported that transection of the hernia sac is linked to a higher incidence of seromas compared to reduction techniques [26], whereas Li et al. discovered no difference between these methods, suggesting hernia sac transection [27]. The use of sutures to obliterate dead space appears ineffective in increasing CPIP rates while reducing seroma occurrences [28]. Additionally, direct closure of hernia defects using barbed suture did not affect chronic pain rates, and peritoneal flap closure has not been linked to increased CPIP [29,30].

Mesh-related considerations are crucial in the context of CPIP development. Biological meshes have not demonstrated any reduction in chronic pain and are associated with heightened seroma and recurrence rates compared to synthetic options [31]. Lightweight meshes have shown no added advantage and may even result in higher recurrence rates when pitted against heavyweight meshes [7,32,33]. The debate over slit versus non-slit meshes continues; Aydin et al. found no difference in outcomes in a retrospective study of 155 patients [34], while Zarras et al., in a larger analysis of 1,028 repairs, noted equal CPIP rates but fewer recurrences with slit meshes [35]. Yildirim et al. highlighted non-slit meshes as being linked with reduced chronic pain [36]. Mesh fixation methods also influence outcomes; research by Meshkati Yazd et al. indicates that no fixation correlates with reduced chronic pain without increasing complication rates [37], while Jiang et al., in a meta-analysis of nine RCTs, found no significant differences in complications or recurrence but noted reduced chronic pain with no fixation in two studies, likely due to less nerve manipulation [38]. Etele et al. [39] concluded that fixation increases chronic pain rates, particularly with traumatic fixation techniques. Among these methods, mechanical fixation has been shown to lead to more chronic pain than glue fixation [40,41]. Fibrin glue, in particular, seems to be better than acrylate glue at minimizing CPIP, possibly due to the heat generated during the application of the latter that could damage nerves [7]. The size of mesh pores is also a debated factor; however, Köckerling et al. found no definitive correlation between pore size and chronic pain in a study of 83,768 patients [42].

Prevention

Risk stratification and preoperative prediction models have the potential to identify patients at high risk for CPIP, enabling cautious management that may lower the incidence of chronic pain. Vad et al. developed a model demonstrating good predictive validity for CPIP risk [43]. Additionally, it stands to reason that addressing modifiable risk factors previously mentioned could reduce chronic pain rates. Patient interventions, such as smoking cessation, weight loss, management of comorbid conditions, improvement of overall health, treatment of BPH, avoiding heavy lifting (over 1,000 kg daily), and managing sleep deprivation and psychological issues, could all contribute to preventing CPIP [1,4,5,7,13,18-21]. Laparoscopic inguinal hernia repair markedly lowers the risk of CPIP and might be recommended for patients considered high-risk [2]. Avoiding mesh fixation or opting for fibrin glue fixation is advised due to their association with reduced chronic pain rates [7,37-41]. Additionally, self-fixating meshes present lower rates of chronic pain compared to staple fixation [44]. Having an experienced surgeon conduct the repair can also mitigate CPIP risk [1,7]. Regarding surgical techniques, fascia transversalis plication in direct inguinal hernias is linked to reduced postoperative pain at 30 days and may lessen chronic pain [45]. However, this finding does not align with the typical CPIP definition at three months. Xu et al. noted that TAPP repair involving preperitoneal space dissection without electrocoagulation results in less pain at three months compared to electrocoagulation [46]. Liu et al. found no differences in chronic pain between monopolar energy and blunt dissection in TEP repair through an RCT [47]. Furthermore, artificial intelligence (AI) could be leveraged to aid CPIP prevention. Several studies indicate that AI models can accurately identify anatomical landmarks - such as vas deferens, gonadal vessels, inferior epigastric vessels, nerves, and dissection layers - in TAPP repair, thereby reducing tissue and nerve damage and lowering CPIP risk [3,48,49].

Limitations

This narrative review examines risk factors and prevention strategies for CPIP following laparoscopic inguinal hernia repair, drawing on various studies that include RCTs, cohort studies, meta-analyses, and reviews. Some risk factors, such as the type of mesh used and hernia sac management, remain contentious, and there is a shortage of studies focusing on CPIP prevention. Therefore, more RCTs are needed to explore additional risk factors, formulate risk stratification and preoperative prediction models, and advance CPIP prevention strategies. Moreover, the existing literature on CPIP exhibits considerable variability and inconsistency. Key aspects of CPIP research, particularly in prevention, such as CPIP definitions, follow-up periods, validated assessment tools, and baseline preoperative measurements, differ widely across studies. This heterogeneity complicates meta-analyses and systematic reviews and hinders the identification of effective prevention strategies [7,50]. A limitation of this review is that it was not prospectively registered in the International Prospective Register of Systematic Reviews (PROSPERO), which may increase the risk of reporting bias. Moreover, the review was originally designed as a narrative synthesis, and a formal risk-of-bias assessment was not conducted. The Grading of Recommendations Assessment, Development and Evaluation (GRADE) was not utilized to formally grade evidence certainty.

## Conclusions

Numerous modifiable risk factors related to CPIP after laparoscopic inguinal hernia repair are highlighted in the literature. By avoiding these risk factors, stratifying patients for CPIP risk, and implementing prevention strategies, the likelihood of CPIP may be significantly reduced, ultimately improving patients' quality of life. Achieving these objectives requires additional RCTs to delve deeper into risk factors and prevention approaches for CPIP while addressing the variability observed in CPIP research.
